# Working with a Patient With Personality Disorder: A Case Report and Reflection on My Experience

**DOI:** 10.1192/bjo.2022.354

**Published:** 2022-06-20

**Authors:** Jiaying Chen

**Affiliations:** Oxford Foundation School, Thames Valley Region, United Kingdom

## Abstract

**Aims:**

Emotionally unstable personality disorder (EUPD) accounts for up to 20% of diagnoses in the inpatient psychiatry population. The assessment, diagnosis, and treatment of any personality disorder may be challenging, and its classification remains debatable. Here I will describe a case of a dual diagnosis of EUPD and schizotypal personality disorder. Through the case report I will also reflect on my first experience of working with a patient with personality disorder, as a Psychiatry Foundation Fellowship doctor with little previous exposure to the psychiatry specialty.

**Methods:**

The patient was a female in her thirties, previously diagnosed with EUPD, who had not benefitted from a number of psychological treatments. She had a history of suicidal behaviour and previous admissions but presented differently this time. She had short hair that was dyed in a vivid colour, was paranoid that she was being spied upon from an alternative universe and had suicidal plans to join the alternative universe. She also had auditory and visual hallucinations. On exploration it became apparent that she had similar episodes in the past, each lasting no more than a day. An additional diagnosis of schizotypal personality disorder was made, and she responded well to risperidone. Unfortunately, she was transferred to another ward for bed management reasons, whereupon the diagnosis reverted to EUPD and antipsychotics were stopped.

**Results:**

This case highlights how in mixed personality disorders, features of one personality disorder may be more predominant than another at different times. It also contradicts the notion that people with schizotypal personality disorder rarely present to mental health services. The inconsistency of diagnosis and lack of continuity of care caused immense distress to the patient, prolonging the acute episode. This highlights the importance of a good formulation in order to tailor care for the patient.

**Conclusion:**

As a newly qualified doctor, working with patients with personality disorders was a meaningful experience. Through ward rounds and the seemingly trivial conversations along the corridor, I thought about the effect of transference and countertransference for the first time, which is applicable to any interpersonal interaction. I witnessed the harm caused by the lack of continuity of care. I reflected on the intricate balance between the advantage of establishing a diagnosis for the patient, and the drawback of the diagnosis leading to labelling. It made me face the stereotypes I held and allowed me to learn about the patient as an individual.

